# PARP Inhibition Attenuates Histopathological Lesion in Ischemia/Reperfusion Renal Mouse Model after Cold Prolonged Ischemia

**DOI:** 10.1155/2013/486574

**Published:** 2013-11-11

**Authors:** Raimundo M. G. del Moral, Mercedes Gómez-Morales, Pedro Hernández-Cortés, David Aguilar, Trinidad Caballero, Jose Aneiros-Fernández, Mercedes Caba-Molina, Mª Dolores Rodríguez-Martínez, Andreina Peralta, Pablo Galindo-Moreno, Antonio Osuna, F. Javier Oliver, Raimundo G. del Moral, Francisco O'Valle

**Affiliations:** ^1^Provincial UGC Intercentre, Department of ICU, Granada, Spain; ^2^Department of Traumatology and Orthopedic Surgery, IBIMER, “San Cecilio” Clinical Hospital and University of Granada, Spain; ^3^Department of Pathology and IBIMER, School of Medicine, University of Granada, Spain; ^4^Institute of Parasitology and Biomedicine, CSIC, Granada, Spain; ^5^Oral Surgery and Implant Dentistry Department, School of Dentistry, University of Granada, Spain; ^6^Department of Periodontics and Oral Medicine School of Dentistry, University of Michigan, Ann Arbor, MI, USA; ^7^Department of Nephrology, “Virgen de las Nieves” Universitary Hospital, Granada, Spain

## Abstract

We test the hypothesis that PARP inhibition can decrease acute tubular necrosis (ATN) and other renal lesions related to prolonged cold ischemia/reperfusion (IR) in kidneys preserved at 4°C in University of Wisconsin (UW) solution. *Material and Methods*. We used 30 male Parp1^+/+^ wild-type and 15 male Parp1^0/0^ knockout C57BL/6 mice. Fifteen of these wild-type mice were pretreated with 3,4-dihydro-5-[4-(1-piperidinyl)butoxyl]-1(2H)-isoquinolinone (DPQ) at a concentration of 15 mg/kg body weight, used as PARP inhibitor. Subgroups of mice were established (A: IR 45 min/6 h; B: IR + 48 h in UW solution; and C: IR + 48 h in UW solution plus DPQ). We processed samples for morphological, immunohistochemical, ultrastructural, and western-blotting studies. *Results*. Prolonged cold ischemia time in UW solution increased PARP-1 expression and kidney injury. Preconditioning with PARP inhibitor DPQ plus DPQ supplementation in UW solution decreased PARP-1 nuclear expression in renal tubules and renal damage. Parp1^0/0^ knockout mice were more resistant to IR-induced renal lesion. In conclusion, PARP inhibition attenuates ATN and other IR-related renal lesions in mouse kidneys under prolonged cold storage in UW solution. If confirmed, these data suggest that pharmacological manipulation of PARP activity may have salutary effects in cold-stored organs at transplantation.

## 1. Introduction

Until now, renal ischemia-reperfusion (IR) injury has been inevitable in transplantation, resulting in the cell death of renal tubular epithelial cells. Human kidneys retrieved from cadaver donors for transplantation are perfused and preserved in order to attenuate ischemic injury between the harvesting of the organ and its transplantation. Currently, the most effective preservation strategy to reduce ischemic injury during prolonged cold storage is to decrease renal oxygen demand by using both hypothermia and preservation solutions (University of Wisconsin (UW) solution) together. These solutions contain adenosine and impermeable saccharides to prevent ATP precursor depletion and cell swelling, respectively. However, this strategy is not sufficiently effective to prevent preservation injury of the tissue [1, 2]. 

Acute tubular necrosis (ATN) induced by IR injury contributes to the development of delayed graft function (DGF), which remains an important complication of kidney transplantation and, despite advances in cold storage, is a major risk factor for acute rejection and long-term kidney allograft dysfunction [3]. Organ preservation solutions play an important role in the prevention of DGF, especially in organs from expanded criteria donors or exposed to prolonged cold ischemia. [4].

The nuclear protein poly(ADP-ribose) polymerase-1 (PARP-1) is a 116 kDa protein member of PARP enzyme family comprised of three functional domains. The amino-terminal DNA-binding domain contains two zinc fingers that are important for the binding of PARP-1 to single-strand breaks and double-strand breaks [5, 6] and, especially, for the detection of DNA breaks by genotoxic and oxygen-derived free radicals. A third zinc finger is important for coupling damage-induced changes in the DNA-binding domain to alterations in PARP-1 catalytic activity [7–9]. PARP-1, known to play a role as DNA damage sensor and in different DNA repair pathways, was recently implicated in a wide variety of cellular functions, including the regulation of transcription [10]. IR injury produces excessive PARP-1 activation (see review of Virág and Szabó, 2002) [11], leading to nicotinamide adenine dinucleotide (NAD+) and ATP depletion, which produce various types of DNA damage [12, 13] that potentially contribute to a type of metabolic cell death designated parthanatos [14]. In a previous study of human biopsies, we demonstrated that PARP-1 expression was significantly related to cold ischemia time, time to effective diuresis, serum creatinine levels, and degree of ATN [15]. 

The objective of the present study in mice was to test the hypothesis that PARP inhibition can reduce ATN and other renal lesions associated with prolonged cold IR in kidneys preserved in UW solution, thereby improving the structural preservation of renal grafts.

## 2. Material and Methods

### 2.1. IR Mouse Model

We used 30 male Parp1^+/+^ wild-type (groups 1 and 2) and 15 male Parp1^0/0^ knockout (group 3) C57BL/6 mice (24 wks old and 20–30 g). Knockout mice were obtained according to a previously reported procedure [16]. 

We kept the mice under stable conditions with *ad libitum* access to food and water. All experiments were performed in a homologated laboratory according to the European Union and Spanish Government guidelines for the ethical care of animals (EU Directive 86/609, RD 223/1988).

Mice were anesthetized by intraperitoneal inoculation of equitensin (2 IU/20 g) and maintained at 37°C on a thermal plate. The left kidney was accessed by anterolateral abdominal horizontal incision of 1.5 cm, and the vascular pedicle was clamped under surgical stereo microscope with a model 2A S&T metallic clip (S&T Microlab AG, Rheinfall, Switzerland), maintaining the kidneys within the abdominal cavity under UW solution flow at 4°C. After 45 min of clamping, the clip was removed and the peritoneum and skin were sutured. After 6 h of reperfusion, the animals were killed with an overdose of sodium pentothal and perfused with cold UW solution by intracardiac injection. Three animals died during postoperative or reperfusion periods and were replaced. 

### 2.2. Administration of PARP Inhibitor to Mice

The PARP inhibitor 3,4-dihydro-5-[4-(1-piperidinyl) butoxyl]-1(2H)-isoquinolinone (DPQ) was purchased from Alexis Biochemicals Corporation (Thermo Fisher Scientific, Waltham, MA) and dissolved in dimethyl sulfoxide (DMSO) at a concentration of 15 mg/kg body weight. DPQ was administered intraperitoneally at 24 h before ischemic injury (as preconditioning) in group 1. Preliminary control experiments had shown that administration of DPQ to sham-operated mice had no morphological effects (data not shown). 

### 2.3. Renal Samples and Processing

Three subgroups (*n* = 5 each) of left kidneys from C57BL/6 Parp1^+/+^ and C57BL/6 Parp1^0/0^ mice were established (A: IR 45 min/6 h immediately followed by euthanasia; B: IR 45 min/6 h then followed by 48 h immersion at 4°C in UW solution; and C: IR 45 min/6 h then followed by 48 h immersion at 4°C in UW solution plus DPQ (15 mg/mL)). The right kidneys served as controls (Parp1^+/+^ *n* = 30; Parp1^0/0^ *n* = 15). Postextraction, each kidney sample was divided transversally into two halves. One half, with cortex and medulla, was rapidly frozen in isopentane at −50°C and immersed in liquid nitrogen for 10 s to develop western blotting. The other half was immediately fixed in 10% buffered formalin for 24 h and then paraffin-embedded for morphological study using hematoxylin-eosin and PAS staining, which was done in blinded fashion on 4-micrometer sections with light microscopy. The presence of acute necrosis, sloughing, and vacuolization of tubular cells was calculated semiquantitatively on a 4-point scale (0, absence; 1, mild (<10% of tubules involved); 2, moderate (10 to 25%); 3, severe (>25%)). The other variables (vascular lesion, glomerular lesion, altered/lost brush border, and tubular cast) were dichotomous (presence/absence). 

### 2.4. Immunohistochemical Analysis

Nuclear expression of PARP-1 was characterized by incubating sections for 30 min at room temperature with PARP-1 monoclonal antibody (clone A6.4.12; Thermo Fisher, Fremont, CA, USA). The immunochemistry study used an automatic immunostainer (model autostainer480, Thermo Fisher) according to the polymer peroxidase-based method, followed by development with diaminobenzidine (Master Diagnóstica, Granada, Spain). The positivity of immunostaining was calculated semiquantitatively on a 4-point scale (0, absence; 1 (1–9% of tubular nuclei positive); 2 (10–49%); 3 (≥50%)). In addition, a millimeter scale in the eyepiece of a BH2 microscope (Olympus Optical Company Ltd, Tokyo, Japan) with ×40 objective was used to count positive nuclei of cortical tubular cells/mm^2^. Results were expressed as positive cells/mm^2^. Renal sections incubated with isotype antibody were used as negative controls. 

### 2.5. Western Blotting

Western blotting was performed according to previously published methods [17]. Tissues extracted from the mice kidneys were washed with PBS and resuspended in 100 *μ*L lysis buffer (50 mM Tris-HCl pH 8, 0.1 mM EDTA, 0.5% Triton X-100, 12.5 mM *β*-mercaptoethanol) for 30 min on ice. Pellet was eliminated and sample buffer (50 mM Tris-HCl pH 6.8, 6 M urea, 6% *β*-mercaptoethanol, 3% SDS, 0.003% bromophenol blue) was added to the supernatant. Proteins were resolved on SDS- 12% polyacrylamide gels and transferred onto Immun-Blot PVDF Membrane (Bio-Rad, Laboratories Irvine, CA). The blot was blocked with 5% milk powder in PBS with 0.1% Tween-20 for 30 min, washed with PBS/Tween, and incubated overnight with anti-poly (ADPribose) (PAR) (TREVIGEN, Gaithersburg, MD), anti-PARP-1 (clone C2-10) (Alexis, San Diego, CA, USA), and anti-*α*-tubulin (Sigma, St Louis MO, USA) antibodies for 2 h with appropriate secondary antibodies. Bands were visualized by ECL-PLUS (Amersham Biosciences, Piscataways, NJ), and photographs were taken with the ChemiDoc XRS imaging system (Bio-Rad).

### 2.6. Electron Microscopy

Several 1 mm² fragments of kidney cortex from each animal were fixed in 2.5% glutaraldehyde solution and then postfixed in 1% OsO_4_ at 4°C for 2 h, washed in distilled water, dehydrated in increasing concentrations of acetone, and embedded in Epon following a conventional protocol. Semithin sections were stained with toluidine blue solution. Ten blocks of LC cluster areas were sampled. Ultrathin (~70 nm-thick) sections were obtained in a Reichert Jung ULTRACUT ultramicrotome (Leica, Westlar, Germany) and stained with lead citrate and uranyl acetate. Ultrathin sections were examined under a Zeiss EM 902 transmission electron microscope and processed using analysis software for Windows (Soft Imaging System, Münstes, Germany).

### 2.7. Statistical Analysis

A descriptive analysis was performed, and the Mann-Whitney *U*-test and Kruskal Wallis test were applied to determine statistical significances. SPSS-Windows 20.0 (IBM SPSS Inc, Chicago, IL, USA) was used for data analyses. The confidence interval was 95% (*P* < 0.05).

## 3. Results

### 3.1. Histopathological Kidney Lesions

**Figure 1:**
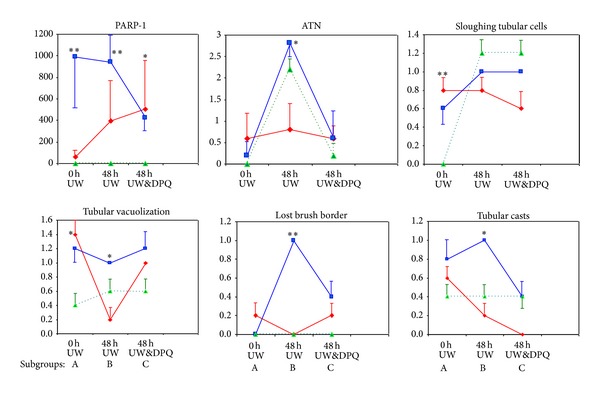
Comparison of PARP-1 expression and kidney lesions between C57BL/6 mouse subgroups (A, B, and C). Red line: Parp1^+/+^ and DPQ (i.p.), group 1. Blue line: Parp1^+/+^ wild-type, group 2. Green line: Parp1^0/0^, group 3. ATN: acute tubular necrosis. *P < 0.05, **P < 0.01 Kruskal Wallis test.

**Figure 2:**
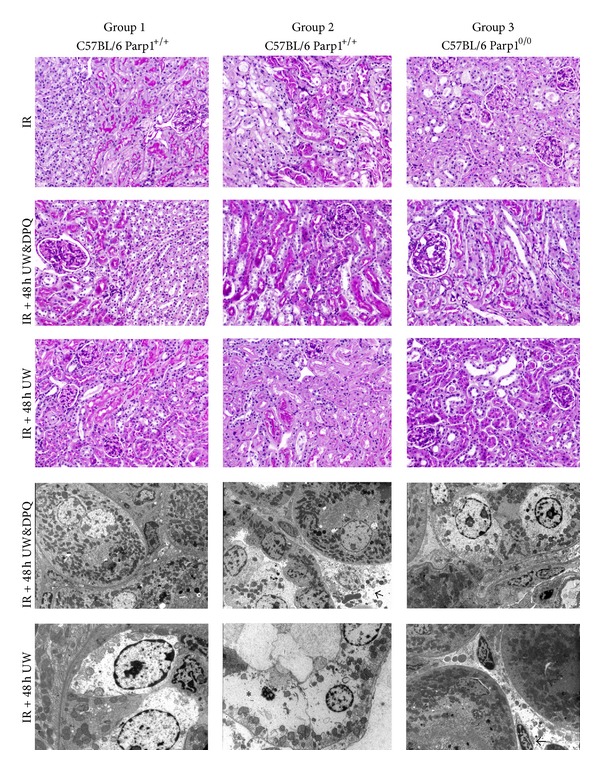
Morphological kidney injury after ischemia/reperfusion (IR) in different groups and subgroups of C56BL/6 mice. (PAS, original magnification 20x). Note loss of brush border and increased acute tubular necrosis in Parp1^+/+^ mouse kidney (IR + 48 h UW subgroup) but only tubular vacuolization in Parp1^+/+^ mouse kidney (IR + 48 h UW&DPQ subgroup). Kidney structure was preserved in all subgroups of Parp1^0/0^ and in Parp1^+/+^ wild-type mice pretreated with ip DPQ. Ultrastructural study confirms higher tubular injury in kidneys immersed for 48 h at 4°C in University of Wisconsin (UW) solution (asterisk). Arrows indicate endothelial cell injury in peritubular capillaries (original magnification ×4600).

Control kidney tissue sections had a normal morphology, with no evident structural changes in tubules, vessels, or glomeruli (Table 1). IR-exposed kidneys showed distinctive patterns of ischemia renal injury that varied in severity among the groups (Figure 1), including widespread degeneration of tubular architecture, alteration/loss of brush border, sloughing of tubular epithelial cells from basement membrane, scant tubular vacuolization, tubular cell necrosis, and intratubular cast formation in the outer medulla (including proximal tubule S3 segment and thick ascending limb). Ultrastructurally, we observed moderate vacuolization of proximal convoluted tubular cells with cytoplasmatic edema and intense injury to endothelial cells in renal peritubular capillaries (Figure 2). Table 1 summarizes the results of comparing histopathological variables among the groups (3 groups of IR kidneys and controls).

### 3.2. Prolonged Cold Ischemia Time in UW Solution Increases PARP-1 Expression and Kidney Injury

**Figure 3:**
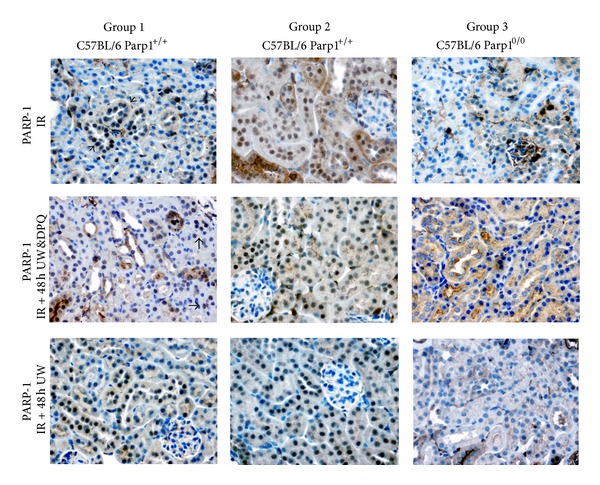
Modification in mouse kidney tubular PARP-1 expression. Kidneys immersed for 48 h at 4°C in UW induces nuclear PARP-1 tubular overexpression, while the DPQ-treated subgroup shows decreased PARP-1 expression (polymer peroxidase-based method, original magnification 20x).

**Figure 4:**
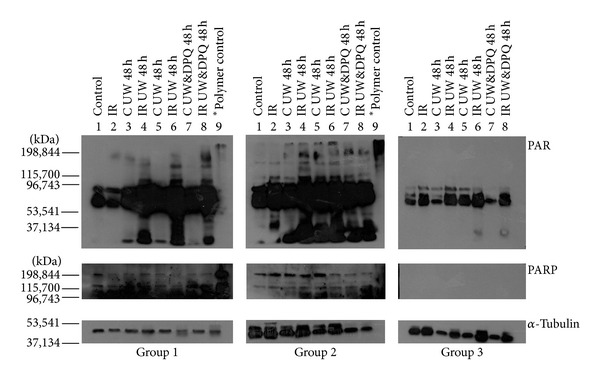
Protein ribosylation was increased in IR kidneys immersed for 48 h at 4°C in UW without DPQ PARP-1 inhibitor; this increase was smaller in subgroups without pretreatment (line 8, group 2) and smallest in IR kidneys immersed for 48 h at 4°C in UW with DPQ supplementation (line 8, group 1).

ATN, sloughing of tubular cells, alteration/loss of brush border, and tubular casts were significantly more evident in kidneys immersed for 48 h in WU (Figures 1 and 2). These lesions were related to higher PARP-1 expression by immunohistochemistry (Figure 3) and to higher PARP expression and greater kidney protein ribosylation by western blotting (Figure 4). 

### 3.3. Preconditioning with PARP Inhibitor DPQ Plus DPQ Supplementation in UW Solution Reduces PARP-1 Nuclear Expression in Renal Tubules

Treatment with i.p. DPQ at 24 h before IR significantly reduced tubular PARP-1 expression, tubular injury (ATN and vacuolization), and the number of animals affected. Kidneys from DPQ-treated animals preserved in UW solution plus DPQ evidenced a greater reduction in PARP-1 expression and tubular injury, finding no significant differences with their respective controls (Table 1 and Figure 1). A reduction in protein ribosylation was also detected by western blotting (Figure 4). The presence of DPQ in WU solution alone (without DPQ pretreatment) also produced a significant decrease in PARP-1 expression and tubular lesions (Figure 3). 

### 3.4. Parp1^0/0^ Knockout Mice are More Resistant to IR-Induced Renal Lesion

The absence of PARP-1 significantly reduced IR-induced tubular lesions, finding scant histopathological alterations and no significant differences with the changes observed in control kidneys (Table 1).

## 4. Discussion

The main finding of this study was that the administration of PARP inhibitor DPQ to kidneys and its addition to the UW solution in which they are cold-stored attenuated ATN and other IR-related lesions. These results, if confirmed, would indicate that the pharmacological manipulation of PARP activity may exert salutary effects in clinical settings in which reperfusion injury is problematic, as in cold-stored organs at transplantation.

After cellular exposure to genotoxic agents in IR injury, the genome integrity of PARP is maintained, but extensive DNA damage may lead to excessive PARP activation, which consumes large quantities of cellular NAD, producing ATP depletion and cell death [18]. Therefore, although chronic inhibition of PARP activity is likely to be harmful to cells, it has been proposed that its transient inhibition after IR injury may prevent cell death [19]. 

Tissue ischemia and reperfusion result in activation of the nuclear enzyme PARP. This enzyme is activated 500-fold by single-strand DNA breaks and cleaves NAD^+^ to attach ADP-ribose polymers to proteins associated with the damaged DNA [20, 21]. Hypoxia due to decreased blood flow leads to a breakdown in cellular energy metabolism and can generate reactive oxygen species (ROS) and reactive nitrogen species [18, 19, 22]. However, our finding of increased PARP-1 expression and activation of protein poly(ADP-ribosyl)ation during cold storage without reperfusion implies that PARP is also activated by other agents besides ROS during the time interval between organ harvesting and transplantation. In a previous immunohistochemistry study by our group, all 20 kidneys were ruled out for transplantation but preserved as whole perfused kidneys that showed a marked increase in PARP-1 expression between the biopsy at 0 h and the renal cortex after 48 h of cold ischemia in UW solution, when a mild activation of PARP-1 was evidenced by western blotting results [15].

Numerous studies have demonstrated a significant role for PARP in reperfusion injury in a variety of tissues, organs, and models. Early investigations demonstrated that chemically distinct inhibitors of PARP activity such as benzamides and isoquinolinones can reduce the degree of injury associated with IR of different organs [22–28], providing the basis for potential clinical applications of PARP inhibitors [29]. Furthermore, the degree of tissue injury caused by IR is attenuated in Parp knockout mice [30].

Isoquinolinones such as DPQ, 1,5-DHIQ [1,5-dihydroxyisoquinoline (5-hydroxyisoquinolin-1(2H)-one)], PJ-34 (phenanthridinone-based PARP inhibitor), INO-1001 (indeno isoquinolinone), and FR247304 are potent PARP inhibitors [30] but are only soluble in solvents such as dimethylsulfoxide (DMSO), which is itself a potent scavenger of hydroxyl radicals and inhibits PARP activity [31]. This explains why DMSO *per se* can reduce IR-related kidney injury [32, 33] without, at the low concentration used, inducing morphological changes.

The lesions observed in this murine IR model correspond to incipient ATN alterations. Their relationship with Parp overactivation is evidenced by the few renal parenchymatous lesions in PARP-1^0/0^ mice and by the reduction in lesions and increase in protein poly(ADP-ribosyl)ation after DPQ administration in parp1^+/+^ wild-type mice. Chatterjee previously reported that PARP activation contributes in part to postreperfusion renal dysfunction and damage to renal tissue with previous ischemia, based on the following experimental evidence: (1) an increased immunohistochemical expression of PARP after renal IR, (2) a significant improvement in renal alterations (reduced urea and creatinine levels and increased glomerular filtrate) with the use of benzamide analogs, selective PARP inhibitors, (3) the lack of effect on IR-induced renal dysfunction of aminobenzoic and nicotinic acids, which do not inhibit PARP [34], and (4) protection by PARP inhibitors of primary cultures of rat proximal kidney tubules against lesion and oxidative stress-mediated cell death [35]. Further research is warranted to confirm our finding of a reduction in renal lesions when DPG is used for preconditioning and also added to the WU solution, which may represent a new strategy to avoid damage during the cold storage of renal grafts. 

## Figures and Tables

**Table 1:** Morphological and immunohistochemical comparison between control and IR C57BL/6 kidney mice samples.

	DPQ treated control group PARP-1^+/+^	DPQ treated IR (group 1)	*P* values MW	Control group PARP-1^+/+^	IR (group 2)	*P* values MW	Control group PARP-1^0/0^	IR (group 3) PARP-1^0/0^	*P* values MW
PARP-1 (mm^2^)	548.64 ± 303.19	320.42 ± 475.34	**0.026**	784.57 ± 353.95	1124.70 ± 550.93	**0.050**	0.0 ± 0.0	0.0 ± 0.0	1
ATN	0.0 ± 0.0	0.93 ± 1.22	0.126	0.0 ± 0.0	1.20 ± 1.42	**0.029**	0.20 ± 0.41	0.80 ± 1.15	0.250
Sloughing tubular cells	0.60 ± 0.51	0.73 ± 0.458	0.539	0.0 ± 0.0	0.87 ± 0.35	**0.000**	0.07 ± 0.26	0.80 ± 0.68	**0.004**
Tubular vacuolization	0.40 ± 0.51	0.64 ± 0.16	0.740	0.27 ± 0.59	1.13 ± 0.52	**0.000**	0.27 ± 0.46	0.53 ± 0.52	0.217
Brush border loss	0.07 ± 0.26	0.13 ± 0.35	0.775	0.0 ± 0.0	0.47 ± 0.52	**0.029**	0.0 ± 0.0	0.0 ± 0.0	1
Tubular casts	0.20 ± 0.41	0.27 ± 0.46	0.775	0.0 ± 0.0	0.73 ±0.60	**0.001**	0.13 ± 0.35	0.40 ± 0.51	0.217

MW: Mann-Whitney *U*-test, DPQ: 3,4-dihydro-5-[4-(1-piperidinyl)butoxyl]-1(2H)-isoquinolinone, IR: ischemia/reperfusion.
